# A construction of 4,4-spirocyclic γ-lactams by tandem radical cyclization with carbon monoxide

**DOI:** 10.3762/bjoc.9.151

**Published:** 2013-07-05

**Authors:** Mitsuhiro Ueda, Yoshitaka Uenoyama, Nozomi Terasoma, Shoko Doi, Shoji Kobayashi, Ilhyong Ryu, John A Murphy

**Affiliations:** 1 Department of Chemistry, Graduate School of Science, Osaka Prefecture University, Sakai, Osaka 599-8531, Japan; 2 Department of Applied Chemistry, Faculty of Engineering, Osaka Institute of Technology, 5-16-1 Ohmiya, Asahi-ku, Osaka 535-8585, Japan; 3 Department of Pure and Applied Chemistry, University of Strathclyde, 295 Cathedral Street, Glasgow G1 1XL, UK

**Keywords:** 4,4-spirocyclic indol γ-lactams, carbon monoxide, free radical, iodoaryl allyl azides, tandem radical cyclization

## Abstract

A straightforward synthesis of 4,4-spirocyclic indol γ-lactams by tandem radical cyclization of iodoaryl allyl azides with CO was achieved. The reaction of iodoaryl allyl azides, TTMSS and AIBN under CO pressure (80 atm) in THF at 80 °C gave the desired 4,4-spirocyclic indoline, benzofuran, and oxindole γ-lactams in moderate to good yields.

## Introduction

**Scheme 1:**
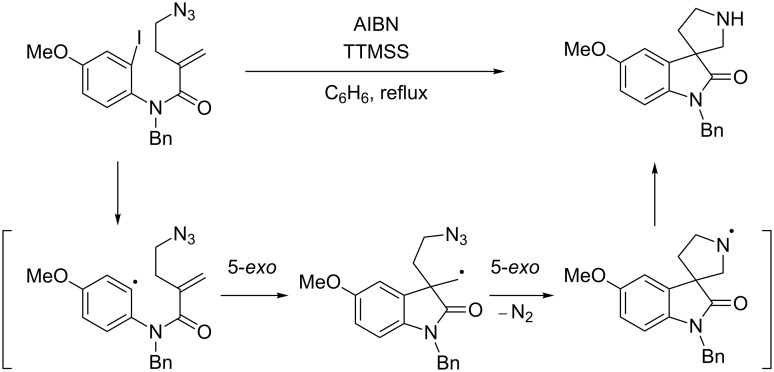
A construction of spirocyclic pyrrolidinyl oxindole by tandem radical cyclization with azide [14].

4,4-Spirocyclic oxindole γ-lactams containing a quaternary carbon center are key structures for the synthesis of biologically active natural products and the related analogues [1–4]. Therefore, the development of an efficient synthesis of this spiro structure is of continued interest for synthetic chemists. Recently, Comesse and Daïch reported the synthesis of 4,4-spirocyclic oxindole γ-lactams by tandem spirocyclization via nucleophilic halide displacement and amide coupling [4]. Shaw and co-workers reported the synthesis of 4,4-spirocyclic oxindole γ-lactams by the cycloaddition of imines and succinic anhydrides [5]. Tandem radical cyclization can also provide a powerful tool for the construction of heterocycles [6–12]. One of us previously reported on the construction of spirocyclic pyrrolidinyl oxindoles by the tandem reaction of iodoaryl alkenyl azides under radical conditions (Scheme 1) [13–14]. Curran et al. reported the synthesis of spirocyclic pyrrolidinyl dihydroquinolinones by tandem radical cyclization [15–16].

**Scheme 2:**
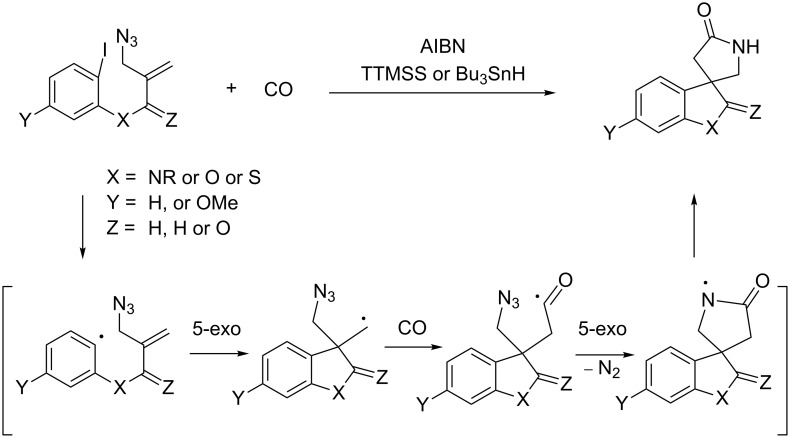
A tandem radical cyclization/annulation strategy for the synthesis of 4,4-spirocyclic γ-lactams with the incorporation of CO.

In this study we report a radical cyclization/annulation approach to 4.4-spirocyclic γ-lactams in which CO was introduced as the lactam carbonyl moiety [17–23]. Our approach consists of a sequence of aryl radical cyclization, radical carbonylation [24–27], and spirocyclization of the resulting acyl radical onto an azide group, which can give 4,4-spirocyclic γ-lactams (Scheme 2).

## Results and Discussion

**Scheme 3:**
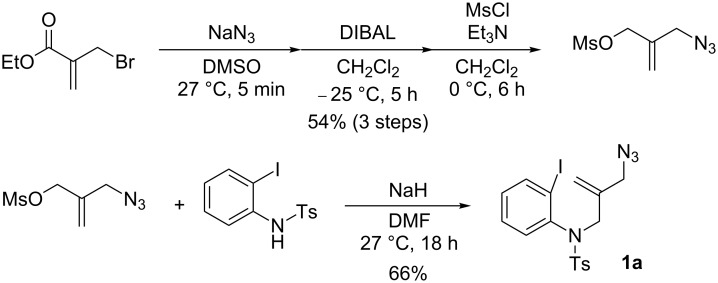
The synthetic methods of **1a**.

**Scheme 4:**
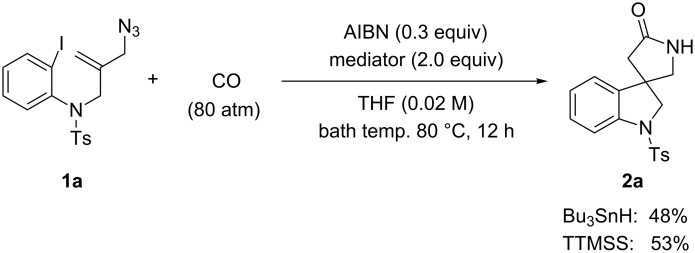
The tandem radical spirocyclization reaction of *N*-(2-(azidomethyl)allyl)-*N*-(2-iodophenyl)-4-methylbenzenesulfonamide (**1a**) with CO.

For the first model reaction in our investigation of the development of a novel tandem radical cyclization/annulation strategy, we prepared *N*-(2-(azidomethyl)allyl)-*N*-(2-iodophenyl)-4-methylbenzenesulfonamide (**1a**) according to the methods shown in Scheme 3. The reaction of **1a** with Bu_3_SnH (2.0 equiv) and AIBN (2,2’- azobisisobutyronitrile, 0.3 equiv) was carried out under CO pressure (80 atm) in THF (0.02 M) at 80 °C for 12 h, which gave the desired 4,4-spirocyclic indoline γ-lactam **2a** in 48% yield (Scheme 4). We found that the modest improvement in the yield of **2a** to 53% was achieved by changing the mediator from Bu_3_SnH to TTMSS [tris(trimethylsilyl)silane].

The tandem spirocyclization with CO was investigated with several 2-iodoaryl compounds having an allyl azide moiety. Results are summarized in Table 1. The reaction of *N*-(2-(azidomethyl)allyl)-*N*-(2-iodo-5-methoxyphenyl)-4-methylbenzenesulfonamide (**1b**) with CO gave the corresponding spiro lactam **2b** in 53% yield (Table 1, entry 2). *N*-(2-(Azidomethyl)allyl)-*N*-(2-iodophenyl)methanesulfonamide (**1c**) showed a comparable reactivity with **1a** and **1b** (Table 1, entry 3). The reaction of 1-(2-(azidomethyl)allyloxy)-2-iodobenzene (**1d**) also gave the spiro benzofuran lactam **2d** in 58% yield (Table 1, entry 4). On the other hand, 2-(azidomethyl)allyl(2-iodophenyl)sulfane (**1e**) gave a low yield of the corresponding spiro thiobenzofuran lactam (19%, Table 1, entry 5), which may be rationalized by the less effective cyclization due to the longer C–S bonds.

**Table 1:** Synthesis of 4,4-spirocyclic γ-lactams **2** by tandem radical spirocyclization of **1** with CO.^a^

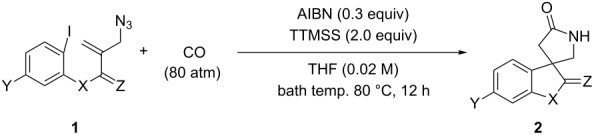			

Entry	Substrate (**1**)	Product (**2**)	Yield (%)

1^b^	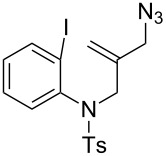 **1a**	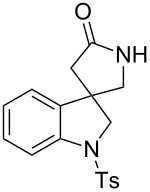 **2a**	53
2^b^	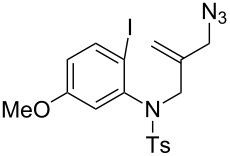 **1b**	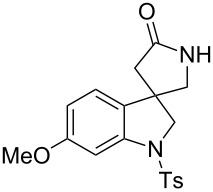 **2b**	53
3	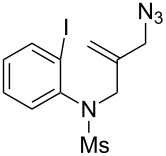 **1c**	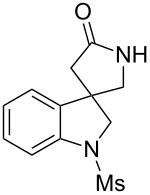 **2c**	55
4	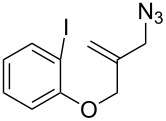 **1d**	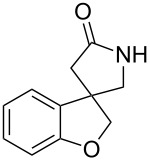 **2d**	58
5^c^	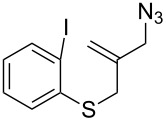 **1e**	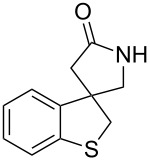 **2e**	19
6	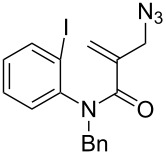 **1f**	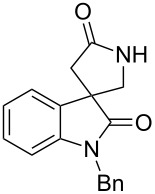 **2f**	62
7	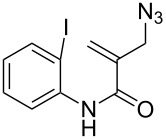 **1g**	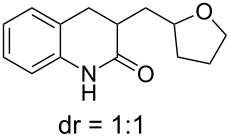 **3**	60^d^

^a^Reaction conditions: **1** (1.0 equiv), CO (80 atm), AIBN (0.3 equiv), TTMSS (2.0 equiv), THF (0.02 M), bath temperature 80 °C, 12 h. ^b^Reaction time: 24 h. ^c^The reaction was carried out at a bath temperature of 110 °C. ^d^Yield of **3**.

We then tried to extend the tandem spirocyclization approach to obtain 4,4-spirocyclic oxindole γ-lactam and tested two substrates, 2-(azidomethyl)-*N*-benzyl-*N*-(2-iodophenyl)acrylamide (**1f**) and the nitrogen-unprotected analogue **1g**. The reaction of **1f** was smooth to give the desired 4,4-spirocyclic oxindole γ-lactam **2f** in 62% yield (Table 1, entry 6). On the other hand, the reaction of **1g** gave the cyclized product in only a trace amount, and instead THF-incorporating 6-*endo* cyclization product **3** was obtained in 60% yield (Table 1, entry 7) [28].

**Scheme 5:**
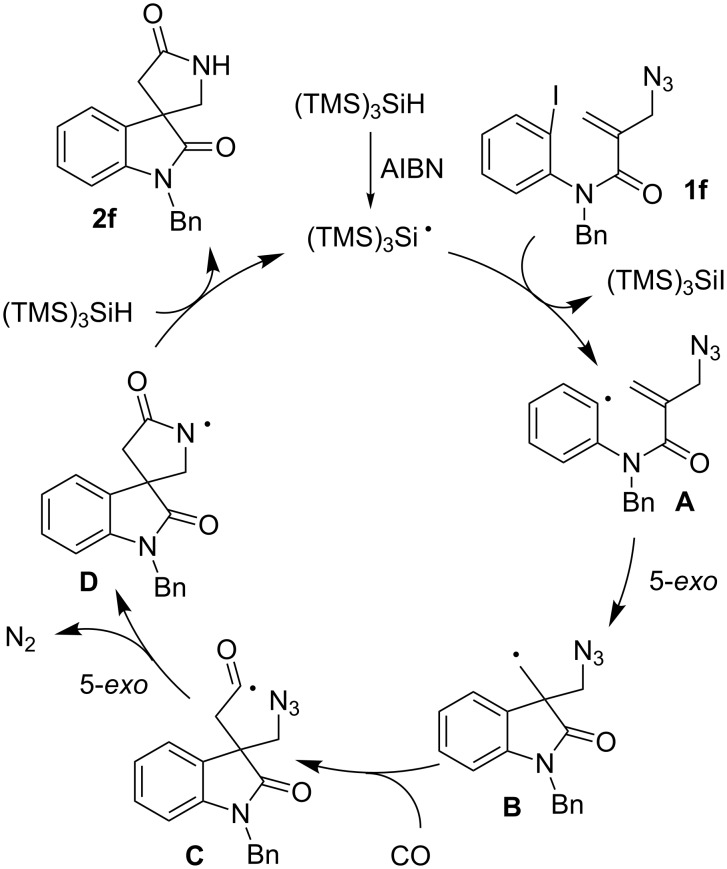
Proposed mechanism for a construction of 4,4-spirocyclic indoline γ-lactam **2f** by the tandem radical cyclization of **1f** with CO.

Based on the known chemistry of radical cyclization and carbonylation reactions, a possible mechanism for the spirocyclization of **1f** with CO is shown in Scheme 5. The iodoaryl allyl azide **1f** is converted to an aryl radical **A** via the iodine atom abstraction by the (TMS)_3_Si radical. The subsequent 5-*exo* cyclization of aryl radical **A** gives an alkyl radical **B**, which adds to CO to give an acyl radical **C**. Finally, the 5-*exo* addition of acyl radical **C** onto an azide group takes place with the liberation of dinitrogen to give a cyclized amidyl radical **D** [29–30], which abstracts hydrogen from TTMSS, affording the 4,4-spirocyclic indoline γ-lactam **2f** and a (TMS)_3_Si radical, thus creating a radical chain.

**Scheme 6:**
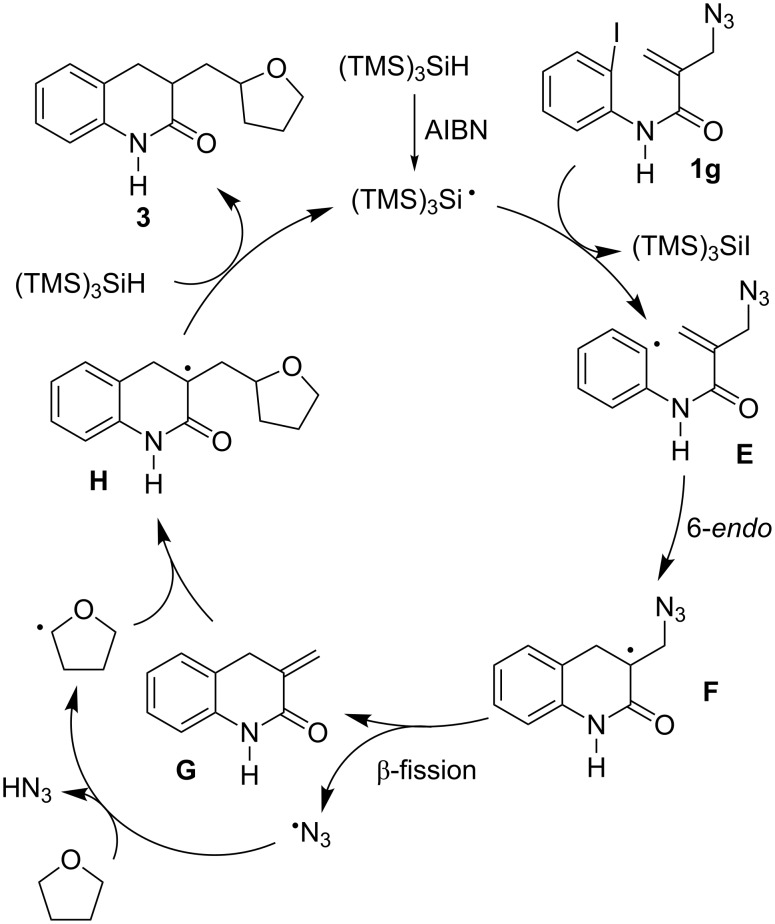
Proposed mechanism for the formation of THF-incorporating product **3** from **1g**.

On the other hand, the unusual formation of THF-incorporating lactam **3** from **1g** may be rationalized by the consecutive 6-*endo* cyclization of **E** and β-elimination of an azidyl radical from the resulting **F**, to give 2-methylene lactam **G** (Scheme 6). Then, the THF radical is formed via the α-hydrogen abstraction by the azidyl radical [31–34], which is attached to **G** to give α-carbonyl radical **H**. Finally, **H** abstracts hydrogen from TTMSS, affording the THF-incorporating product **3** and the (TMS)_3_Si radical, which participates in the next chain reaction.

## Conclusion

We have examined a TTMSS-mediated 5-*exo* radical cyclization/carbonylation/spirocyclization sequence to synthesize 4,4-spirocyclic rings. By using this protocol, indoline, benzofuran and oxindole γ-lactams can be conveniently prepared in moderate to good yields. As shown in the contrasting results of acrylic amides **1f** and **1g**, to cause the requisite 5-*exo* cyclization of aryl radicals onto allylic azide in preference to the 6-*endo* cyclization, the angle compression caused by the substitution on the nitrogen has to be considered carefully. Nevertheless, our method can provide a steady tool for the ring formation of 4,4-spirocyclic γ-lactams with the incorporation of CO as a carbonyl group.

## Experimental

**Typical procedure for a construction of 4,4-spirocyclic γ-lactams by tandem radical cyclization with CO:** A magnetic stirring bar, 2-(azidomethyl)-*N*-benzyl-*N*-(2-iodophenyl)acrylamide (**1f**) (150.0 mg, 0.36 mmol), AIBN (2,2’-azobisisobutyronitrile, 17.7 mg, 0.11 mmol), TTMSS ([tris(trimethylsilyl)silane], 178.3 mg, 0.72 mmol) and THF (17.9 mL; 0.02 M) were placed in a 50 mL stainless steel autoclave. The autoclave was closed, purged three times with CO, pressurized with 80 atm of CO, and then heated at 80 °C (bath temperature) for 12 h. Excess CO was discharged after the reaction. The reaction mixture was concentrated in vacuo*.* The resulting residue was purified by column chromatography on silica gel (hexane/EtOAc 2:1) to give the desired 4,4-spirocyclic oxindole γ-lactam product **2f** as a colorless oil in 62% yield (65.3 mg, 0.22 mmol). ¹H NMR (400 MHz, CDCl_3_) δ 7.39–7.16 (m, 7H), 7.07 (t, *J* = 7.6 Hz, 1H), 6.79 (d, *J* = 7.6 Hz, 1H), 5.89 (s, 1H), 4.93 (s, 2H), 3.91 (d, *J* = 9.2 Hz, 1H), 3.50 (d, *J* = 9.2 Hz, 1H) 3.02 (d, *J* = 16.8 Hz, 1H), 2.51 (d, *J* = 16.8 Hz, 1H); ^13^C NMR (100 MHz, CDCl_3_) δ 177.5, 175.4, 141.9, 135.5, 133.2, 129.1, 129.0, 128.0, 127.4, 123.6, 122.3, 109.7, 51.1, 49.7, 44.3, 40.4; IR (neat): 3418, 3061, 2927, 1696, 1613, 1488, 1467, 1455, 1380, 1368, 1177 cm^−1^; HRMS–FAB (*m*/*z*): [M + H]^+^ calcd for C_18_H_17_N_2_O_2_, 293.1290; found, 293.1299.
